# Real‐World Data of Comprehensive Cancer Genomic Profiling Tests Performed in the Routine Clinical Setting in Sarcoma

**DOI:** 10.1002/cam4.71098

**Published:** 2025-08-04

**Authors:** Eiji Nakata, Daisuke Ennishi, Tatsunori Osone, Kiichiro Ninomiya, Shuta Tomida, Takuto Itano, Tomohiro Fujiwara, Toshiyuki Kunisada, Naoyuki Ida, Hideki Yamamoto, Mashu Futagawa, Tatsunori Shimoi, Hiroyuki Yanai, Akira Hirasawa, Shinichi Toyooka, Masahiro Tabata, Toshifumi Ozaki

**Affiliations:** ^1^ Department of Orthopaedic Surgery Okayama University Graduate School of Medicine, Dentistry, and Pharmaceutical Sciences Okayama Japan; ^2^ Center for Comprehensive Genomic Medicine Okayama University Graduate School of Medicine, Dentistry, and Pharmaceutical Sciences Okayama Japan; ^3^ Department of Regenerative Science Okayama University Graduate School of Medicine, Dentistry, and Pharmaceutical Sciences Okayama Japan; ^4^ Department of Obstetrics and Gynecology Okayama University Graduate School of Medicine, Dentistry, and Pharmaceutical Sciences Okayama Japan; ^5^ Department of Clinical Genomic Medicine Okayama University Graduate School of Medicine, Dentistry, and Pharmaceutical Sciences Okayama Japan; ^6^ Department of Medical Oncology National Cancer Center Hospital Tokyo Japan; ^7^ Department of Pathology Okayama University Graduate School of Medicine, Dentistry, and Pharmaceutical Sciences Okayama Japan; ^8^ Department of General Thoracic and Breast and Endocrinological Surgery Okayama University Graduate School of Medicine, Dentistry, and Pharmaceutical Sciences Okayama Japan; ^9^ Center for Clinical Oncology Okayama University Graduate School of Medicine, Dentistry, and Pharmaceutical Sciences Okayama Japan

**Keywords:** comprehensive genomic profiling, genotype‐matched therapy, multiplex gene panel test, sarcoma

## Abstract

**Introduction:**

Next‐generation sequencing‐based comprehensive cancer genomic profiling (CGP) tests are beneficial for refining diagnosis and personalized treatment of various cancers. However, the clinical impact of CGP, as covered by public health insurance in the management of sarcomas, remains unknown. Especially, the data on the utility of the newly emerging dual DNA–RNA panel compared to the conventional DNA‐only panel in clinical settings is lacking. Therefore, we evaluated the utility of CGP in routine clinical practice for sarcoma treatment.

**Patients and Methods:**

In this study, three types of DNA panel and one DNA–RNA panel, reimbursed by Japanese public health insurance, were utilized. We detected oncogenic and druggable gene mutations and genotype‐matched therapies.

**Results:**

One hundred and thirty‐six patients were included in this study. Based on the detection of highly histology‐specific translocations in the sequencing results, 2.2% of patients were re‐classified. In patients with translocation‐related sarcomas, a DNA–RNA panel identified more histology‐specific fusion genes than DNA panels (*p* = 0.0035). Specifically, 86.8% and 39.0% of patients had oncogenic and druggable genomic alterations, respectively. Of these, 9.6% underwent genotype‐matched therapy, with a 36.3% response rate and an 81.8% disease control rate. Patients who were administered genomically matched therapy had better overall survival (OS) than those who did not in patients with metastatic or advanced sarcoma with no prior chemotherapy (3‐year OS: 83.3% vs. 48.0%, *p* = 0.42). Patients with *TP53* and *RB1* mutations had worse OS than those without. Germline findings were detected in 11.0% of the patients, one of whom had a truly germline origin.

**Conclusions:**

This study suggests that publicly reimbursed CGP tests, particularly the dual DNA–RNA panel, could be beneficial for refining diagnostic precision in selected sarcoma subtypes, treatment decisions, detecting the germline findings, and prognosis prediction in routine clinical settings for sarcoma. The implementation of genotype‐matched therapies showed favorable clinical outcomes and improved the prognosis.

## 1 Introduction

Next‐generation sequencing (NGS)‐based comprehensive cancer genomic profiling (CGP) tests enable refining diagnosis and personalized treatment of various cancers [1, 2, 3, 4, 5, 6, 7, 8, 9]. They can detect mutated genes, which can be potential targets for genotype‐matched therapy, and reduce preventable treatments in patients with cancer [4, 5, 6, 7].

In Japan, CGP testing was introduced in 2019; currently, five CGP tests are reimbursed by the Japanese public health insurance system [10]. The Japanese public health insurance system provides universal coverage for nearly all residents, with most medical costs (typically 70%) covered and the remainder paid by patients. The first four panels were DNA‐based and can detect 124–309 gene alterations and 13–36 gene rearrangements. Although the identification of fusion genes is crucial in some malignancies, such as sarcomas, it is difficult to identify them using the available panels. In 2024, a new insurance‐covered CGP test called GenMine TOP Cancer Genome Profiling System (GenMine TOP) was introduced [11, 12, 13, 14]. GenMine TOP is a novel concept‐based panel test that integrates and analyzes multi‐omics data, including DNA, RNA, and gene expression. This test can detect 737 gene alterations and 455 gene rearrangements and functions as a paired tumor‐normal matching test [14].

Sarcomas are rare cancers that account for 1%–2% of all malignancies [15]. Sarcomas are rare and heterogeneous; therefore, diagnostic errors in sarcomas are common, with inaccuracy rates of up to 10.5% [1]. Some sarcomas have specific molecular characteristics, such as *SYT::SSX* fusion in synovial sarcoma, *EWSR1::FLI1* fusion in Ewing's sarcoma (ES), and *MDM2* amplification in dedifferentiated liposarcoma (DDLS), leading to correct diagnosis and treatment [2]. For unresectable or metastatic sarcomas, therapeutic options mainly rely on systemic therapy. However, in most sarcomas, cytotoxic drugs, including anthracycline‐based chemotherapy, are used, which have limited efficacy and result in poor outcomes.

Several studies have been conducted on CGP in sarcoma [1, 2, 3, 4, 5, 6, 7, 8, 9]. The proportions of potentially actionable alterations and patients who were administered drugs targeting an alteration detected by CGP were 32%–62% and 8%–16%, respectively [1, 5]. However, these reports were based on data from various sequencing platforms, including non‐commercial or in‐house targeted sequencing platforms, in which the genomic profiles were analyzed and annotated at a single institution. Furthermore, the clinical performance of dual DNA–RNA panels, particularly in routine settings, has not yet been evaluated in patients with sarcoma. Additionally, data on the utility of performing CGP tests to improve prognosis is lacking. Therefore, we evaluated the potential clinical impact of CGP, which is covered by public health insurance, on the management of patients with sarcoma.

## 2 Patients and Methods

### 2.1 Study Population

We retrospectively evaluated the records of patients with sarcoma who underwent CGP and were covered by public health insurance between November 2019 and October 2024 at our hospital. We excluded patients with a diagnosis of other malignancy and benign tumor.

### 2.2 Histopathology

Pathologists established histological diagnosis according to the classification of tumors by the World Health Organization [15]. Histologic subtypes were classified into three categories based on their known molecular characteristics: translocation‐related sarcomas (TRS), sarcomas with specific reciprocal translocations resulting in oncogenic fusion transcripts such as synovial sarcoma and myxoid/round cell liposarcomas; genomically complex sarcomas, sarcomas displaying multiple, complex karyotypic abnormalities with no specific pattern; and other sarcomas, which have specific oncogenic mutations or recurrent amplification (Table S1) [1].

### 2.3 Comprehensive Genomic Profiling Tests

In this study, four CGP tests reimbursed by Japanese public health insurance were utilized (Table S2): FoundationOne CDx cancer genome profiling (F1CDx, Chugai Pharmaceutical) and OncoGuide NCC Oncopanel System (NCC Onco‐panel, Sysmex Corporation, Hyogo, Japan) from November 2019, FoundationOne Liquid CDx cancer genome profiling (F1LCDx; Chugai Pharmaceutical, Tokyo, Japan) from August 2021, and GenMine TOP (GenMine, Konica Minolta REALM Inc. Tokyo, Japan) in August 2023. The gene lists analyzed by each CGP test are shown in Table S3. F1CDx, NCC Onco‐panel, and F1LCDx are DNA panels. F1CDx carries 324 genes and determines single nucleotide variants (SNV), insertions/deletions mutations (indels), copy‐number variation (CNV) (amplifications and gains) of 309 genes, fusions of 36 genes, microsatellite instability (MSI) and tumor mutation burden (TMB) [16]. The NCC Onco‐panel carries 114 genes and determines base substitutions, insertion/deletion mutations, gene amplification of 114 genes, fusions of 12 genes, and TMB [17]. GenMine TOP is a dual DNA–RNA panel. It is designed to identify 737 gene alterations (SNV, indels, and CNV (amplifications)) with its DNA panel, while simultaneously detecting 455 fusion transcripts, exon skipping of 5 genes, and gene expression of 27 genes with its RNA panel [11, 12, 13, 14]. The identified fusion genes include almost all fusion genes reported in bone and soft tissue tumors. The RNA panel additionally isolates RNA from formalin‐fixed paraffin‐embedded (FFPE) tumor samples, converts it to cDNA, and employs targeted probes to capture specific regions. This approach enables the detection of fusion mRNA without the need to design multiple probes for extensive intronic areas, which frequently contain repetitive sequences that are difficult to map. The GenMine TOP reports CNV, specifically amplifications, but does not include information on copy number losses. Additionally, the panel does not provide results for MSI status. The NCC Onco‐panel and GenMine TOP function as paired tumor‐normal matched tests. Genomic DNA was isolated from peripheral blood samples as a control reference, allowing for the distinction between somatic and germline alterations. Further technical information regarding the CGP assay and bioinformatic analysis is found in previous studies [11, 12, 13, 14, 17, 18].

The pathologic diagnosis of each case was confirmed by an expert pathologist in review of hematoxylin and eosin–stained slides. When tissue specimens were used, a pathologist determined whether they could be submitted for CGP testing based on the tumor area, tumor content, and specimen storage period. DNA quantity and quality were measured and confirmed to meet the minimum requirements for each panel assay, ensuring suitability for CGP analysis. For F1CDx, NCC Onco‐panel, and the GenMine TOP, specimens needed to have tumor cell proportions of at least 20%. Genomic DNA was extracted from FFPE tumor tissue when the tissue volume or tumor percentage was sufficient. Re‐biopsy was performed in case the samples were not appropriate for CGP. F1LCDx was utilized if tissue specimens were not available.

### 2.4 Expert Panel for Cancer Genomic Profiling Test

The Center for Cancer Genomics and Advanced Therapeutics (C‐CAT) was established at the National Cancer Center to centralize genomic and clinical data from patients undergoing CGP covered by public health insurance in Japan [10, 19]. Drawing from a database of over 90,000 patients in C‐CAT, individual genomic profiles of patients are analyzed and annotated to produce reports recommending personalized treatments based on each patient's genomic profiles. The patient's clinical data and CGP results were gathered to C‐CAT, and pathogenicity for each identified variant was annotated based on the patient's report from the C‐CAT guidelines by the Joint Consensus of the Japanese Society of Medical Oncology (JSMO), Japan Society of Clinical Oncology (JSCO), and Japanese Cancer Association (JCA) [15, 20, 21]. Genomic alterations were classified into seven tiers (A to F, and R) of evidence‐level classifications (Table S4) [16]. C‐CAT report with the information including evidence levels for therapeutic efficacy of agents against genomic alterations, the availability of the therapeutic agents, and the patient's genotype‐matched clinical trial was sent to our hospital. The time between shipping samples to the expert panel was calculated as the turnaround time (TAT). The results of all CGP tests were reviewed by molecular tumor boards (MTB) called ‘expert panel’ consisted of multidisciplinary teams of specialists in cancer genomic medicine at our institution, including oncologists for each organ, clinical geneticists, certified genetic counselors, pathologists, bioinformaticians, and pharmacists. As previously described, potentially oncogenic gene alterations and druggable gene alterations were defined as alterations at or above evidence level F (gene abnormality known to be involved in cancer) and D (biomarker associated with efficacy in a few case reports), respectively [16]. Based on the patient's treatment history, the patient's background, the level and details of the evidence of C‐CAT report, and accessibility of drugs, real‐world oncogenic and druggable gene alterations were determined by the expert panel. The recommendation of genotype‐matched therapy was also determined by the expert panel and then proposed to the patients [20].

### 2.5 Profiling of Genomic Alterations

Single nucleotide variants (SNVs), insertions/deletions (indels), and copy number variations (amplifications and gains) were investigated. Variants of unknown significance and synonymous single‐nucleotide polymorphisms were excluded in this study.

### 2.6 Oncoplot of Reported Oncogenic Mutations

We generated an oncoplot using the Julia package “CairoMakie” (version 0.10.4 [GitHub, San Francisco, CA, USA]) to visualize the landscape of genetic alterations identified by CGP tests. The alterations included SNVs and indels.

### 2.7 Fusion Genes

The identified fusion genes were confirmed by searching five websites: fusionGDB2, TCGA fusion gene database, fusion profiling interactive analysis, mitelman database of chromosome aberrations and gene fusions in cancer, and the COSMIC Fusions database [22, 23, 24, 25, 26]. Additionally, articles published in PubMed were used [27]. A fusion reported in at least one case of the same morphological soft tissue sarcoma (STS) subtype was defined as recurrent, whereas that not reported in the same morphological STS subtype was defined as new [2]. The identified fusion genes were visualized using a Circos plot using the R package “circlize” (version 0.4.16 [RStudio, Boston, MA, USA]).

### 2.8 Tumor Mutation Burden and Microsatellite Instability

High tumor mutation burden (TMB‐H) was defined as a TMB value ≥ 10 mut/Mb [28]. Microsatellite (MSI) scores were classified as MSI‐high (MSI‐H) or microsatellite‐stable.

### 2.9 Evaluation of Germline Findings

Germline findings consisting of presumed germline pathogenic variants (PGPVs) or germline pathogenic variants (GPVs) were obtained from genes with pathogenic variants detected using CGP testing according to recommendations from previous studies [29, 30]. The major factors to consider when detecting PGPVs are tumor purity and variant allele frequency with or without clinical backgrounds, such as the present and/or past histories of specific cancer types and their family histories.

### 2.10 Assessment of Study Outcomes

#### 2.10.1 Statistical Analysis

We determined the relationship between gene alterations, including *TP53*, *RB1*, and cyclin‐dependent kinase inhibitor 2A (*CDKN2A*), the detection of druggable and oncogenic gene mutations, and genotype‐matched therapy. This analysis considered various factors such as generation (pediatric/adolescent and young adult (AYA), middle‐aged/older adult), sex, sampling site (primary tumor or not), molecular characteristics (TRS or others), and tissue of origin (bone or soft tissue). RECIST v1.0 criteria (GitHub) are applied using computed tomography scans taken at least 6 weeks after the initiation of treatment for patients who received genotype‐matched therapy [31, 32]. The best overall response was defined as the best response recorded from the start of the treatment until disease progression [31]. Progression‐free survival (PFS) was defined as the time from the date of CGP testing to disease progression or death from any cause after treatment initiation. Patients alive at the time of analysis were censored on the date of the last disease assessment [31]. The OS rate was calculated from the date of CGP testing to the date of death from any cause or last follow‐up visit. We determined the association between OS and gene alterations (*TP53, RB1*, and *CDKN2A*) in patients with metastatic or advanced sarcoma with no prior chemotherapy. We also estimated the association between OS and the administration of genotype‐matched therapies in them. Fisher's exact test was used to analyze categorical parameters. Survival curves were plotted using the Kaplan–Meier method. Differences in survival rates were assessed using Cox proportional hazards regression analysis. All statistical analyses were performed with EZR (Jichi Medical University, Tochigi, Japan), which is a graphical user interface for R (The R Foundation for Statistical Computing, Vienna, Austria) associations were considered significant at *p* < 0.05.

## 3 Results

### 3.1 Patient Characteristics

This study included 136 patients (62 men, 74 women) (Table S5). Their median age at the time of genomic profiling was 55 years (range, 8–80). There were 5, 23, 62, and 46 patients in pediatric (0–14 years), adolescent and young adult (AYA) (15–39 years), middle‐aged adult (40–64 years), and older adult (> 65 years), respectively. There were 110 STS and 26 bone sarcoma cases. The location of the primary tumor was shown in Table S1. In STS, DDLS was the most common diagnosis, accounting for 19 patients, followed by uterine leiomyosarcoma (ULMS) in 17 patients and leiomyosarcoma (LMS) in 12 patients (Table S6). Osteosarcoma was the most commonly diagnosed bone sarcoma, accounting for 11 patients, followed by chondrosarcoma (CS) in 5. Thirty‐six (26.4%) patients had TRS, and 100 (73.6%) had genomically complex and other sarcomas. F1CDx, NCC Oncopanel, GenMine TOP, and F1LCDx were used in 118 (86.8%), 4 (2.9%), 12 (8.8%), and 2 (1.5%) patients, respectively. Regarding F1CDx, NCC Oncopanel, and GeneMine TOP, FFPE tumor samples were collected from archived specimens; 75, 15, and 44 specimens were collected from primary sites, local recurrent site, and metastatic sites. Submitted specimens included 103 surgery, 30 biopsy, and 1 cell block from pleural effusion. The availability of appropriate tumor specimens was checked, and a re‐biopsy was performed on 5 patients whose samples were not appropriate for the CGP test. The median TAT was 26 days (range: 19–48).

### 3.2 Re‐Classifications of Diagnosis

Based on the detection of highly histology‐specific translocations in the sequencing results, three (2.2%) patients were re‐classified. An initial diagnosis of sarcoma NOS in the abdomen and trunk was classified as ES and extraskeletal myxoid chondrosarcoma (EMC) based on the fusion genes *EWSR1::FLI1* and *NR4A3::EWSR1*, respectively. Other patients with an initial diagnosis of low‐grade fibromyxoid sarcoma were classified as having sclerosing epithelioid fibrosarcoma based on the fusion gene *EWSR1::CREB3L1*.

### 3.3 Profiling of Genomic Alterations

**FIGURE 1:**
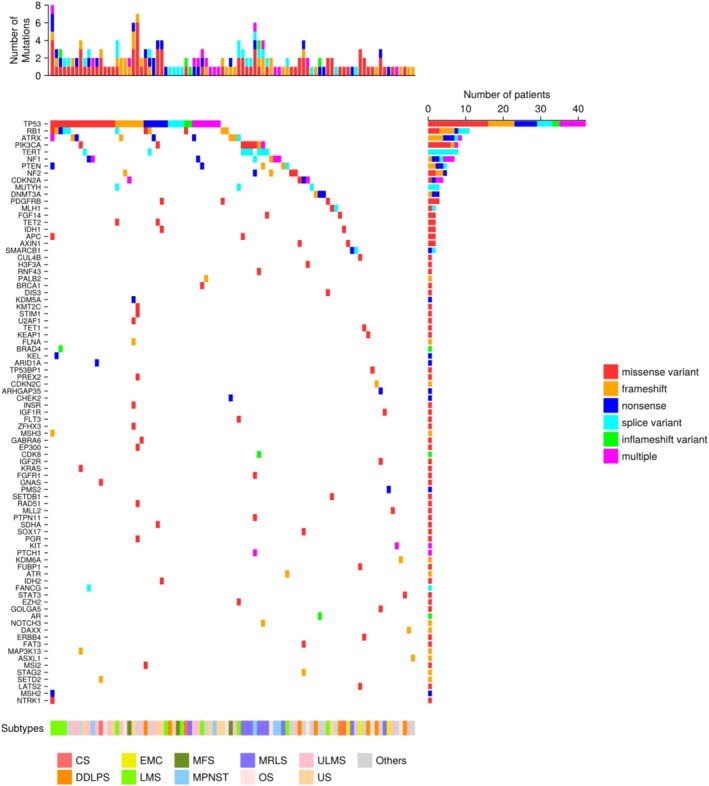
Oncogenic gene alteration by subtype. Oncoplot of oncogenic genomic alterations (SNV/Indels) identified in the study population. Oncogenic genomic alterations in specific genes detected at a frequency > 2 cases are shown. Tumor subtypes are represented in colored text below Oncoplot. The histogram on top of the mutation Oncoplo represents the number of mutations per patient. The mutation frequency of each gene is shown on the right side of the Oncoplot.

Four hundred and thirty‐six genetic variants were identified in 148 genes (Table S7). Furthermore, 124 (91.2%) patients had at least one detectable gene alteration, with a median of one variant per patient (range, 0–8). Among these, SNVs/indels were the most frequent (65.4%, 89 patients), followed by the copy number variant (CNV) (58.8%, 80 patients). The SNVs/indels are presented in the Oncoplot in Figure 1. The most frequently mutated genes were *TP53* (42 patients, 30.9%), followed by *RB1* (11 patients, 8.1%), *ATRX* (9 patients, 6.6%), *PIK3CA* (8 patients, 5.9%), *TERT* (8 patients, 5.9%), *NF1* (7 patients, 5.1%), *PTEN* (5 patients, 3.7%), and *NF2* (5 patients, 3.7%) in SNVs/indels, while cyclin‐dependent kinase 4 (*CDK4*) (22 patients, 16.2%), *CDKN2A* (20 patients, 14.7%), *MDM2* (18 patients, 13.2%), and *CDKN2B* (17 patients, 12.5%) in CNV. The factors contributing to *TP53* alteration include generation, molecular characteristics, and originating tissue, while *RB1* alteration is influenced by sex and molecular characteristics, and *CDKN2A* alteration is associated with molecular characteristics (Tables [Link], [Link], [Link]). The frequency of alterations differed between TRS and complex sarcomas in *TP53* (17.9% vs. 41.7%, *p* < 0.001), *RB1* (2.8% vs. 26.0%, *p* = 0.0015), and *CDKN2A* (5.6% vs. 9.0%, *p* = 0.039) (Tables [Link], [Link], [Link]). The mutational landscapes of sarcoma subtypes were subsequently analyzed. The most commonly mutated genes were *CDKN2A/B* in osteosarcoma, *TP53*, *RB1*, and *ATRX* in ULMS, and *TP53*, *RB1*, and *PTEN* in LMS. Subtype‐specific gene alterations have been identified in liposarcomas, including DDLS and myxoid/round cell liposarcoma (MRCLS). The PI3K pathways (*PIK3CA* and *PTEN*) were frequently altered in MRCLS (7/10 patients), whereas only 2/19 patients demonstrated alterations in DDLS. Contrastingly, co‐amplification of the cell cycle (*CDK4*) or TP53 pathway (*MDM2*) was observed in 16/19 of patients with DDLS, although no MRCLS was observed in patients.

**FIGURE 2:**
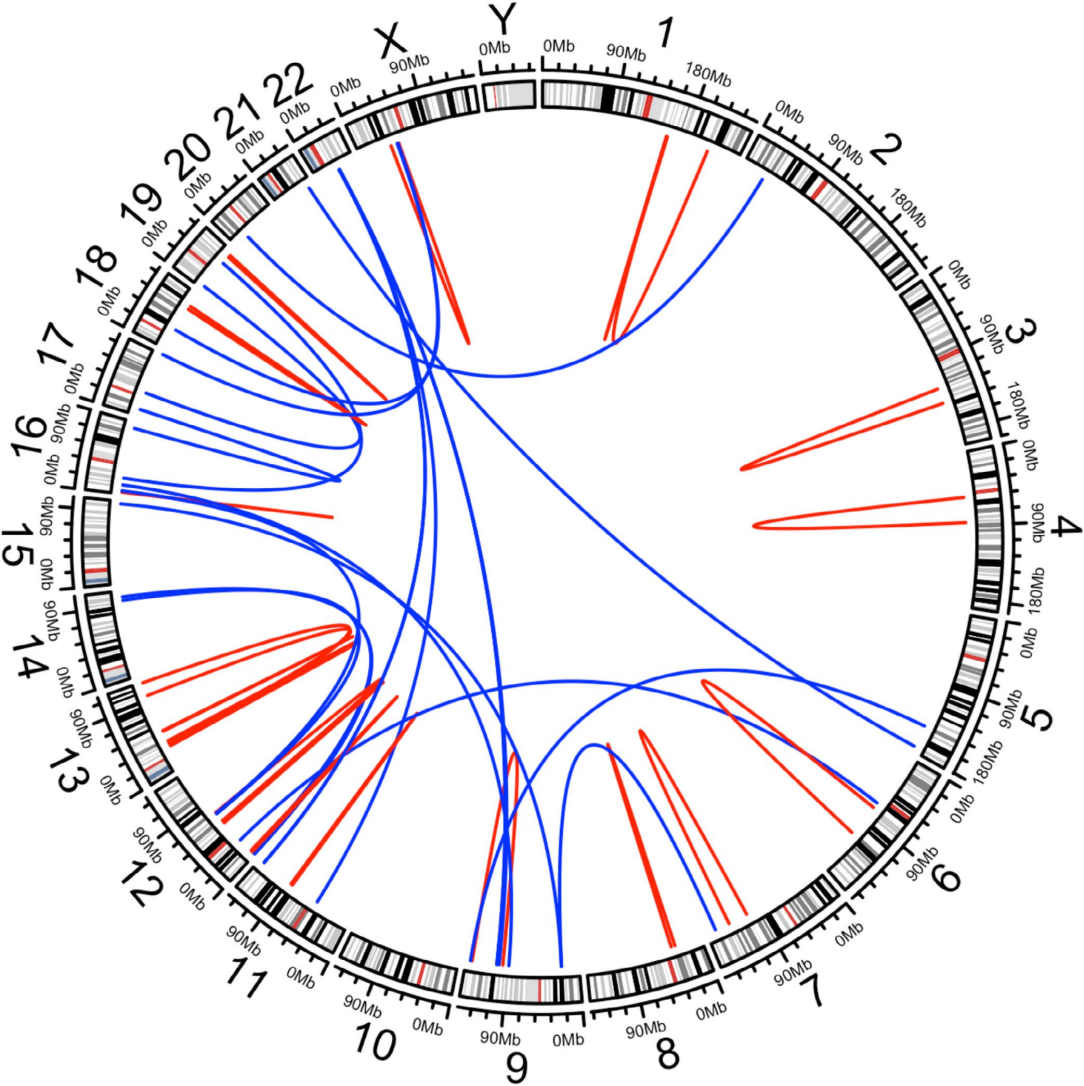
Circos plot of identified fusion genes. The identified fusion genes were visually represented in the Circos plot which indicate both intra‐and inter‐chromosomal rearrangements.

Forty‐two fusion genes were observed in 31 cases (22.8%); 10 cases exhibited 2 fusion genes, and 1 case exhibited 3 fusion genes (Table 1). Fusion genes were detected in 26/118 F1CDx, 0/4 NCC Oncopanel, 5/12 GenMine TOP, and 0/2 F1LCDx strains. Nine fusion genes were specific to particular histotypes, including *SYT::SSX* fusion in synovial sarcoma, *EWSR1::FLI1* fusion in ES, *EWSR1::NR4A3* fusion in EMC, *BCOR::CCNB3* fusion in sarcoma with *BCOR* genetic alterations, and *EWSR1::CREB3L1* fusion in sclerosing epithelioid fibrosarcoma. Among patients with TRS, fusion genes specific to histology were identified in 5 of 6 cases by GenMine TOP and in 5 of 30 cases by the other panels (*p* = 0.0035) (Table S11). Potentially therapeutically targetable kinase fusions were identified in 6 patients (4.4%), including *NTRK* fusion in 3 cases (1 each of OS, DDLS, and ULMS), *ALK* fusion in 1 case (inflammatory myofibroblastic tumor [IMT]), *ROS1* fusion in 1 case (DDLS), and *PDGFRA* fusion in 1 case (undifferentiated pleomorphic sarcoma [UPS]). The 33 novel fusions were identified in 21 patients involving RB1, BCL2, and BRCA2; none were identified in patients with TRS, and 21 (21.0%) were identified in patients with genomically complex and other sarcomas (*p* < 0.001). The identified fusion genes are presented in a Circos plot, which indicates intra‐ and inter‐chromosomal rearrangements (Figure 2).

**TABLE 1:** Gene Fusions in sarcoma subtypes.

Characteristics	Histology	Recurrent fusion	Novel fusion
Soft tissue	Alveolar soft part sarcoma	*ASPSCR1::TFE3* (G)	
	Sarcoma with *BCOR* genetic alterations	*BCOR::CCNB3* (G)^c^	
	Dedifferentiated liposarcoma		*CCND2::SETD3* (F) *CREBBP::RAB3IP* (F) *DICER1::MDM2* (F) *EED::DLG2* (F) *ERBB3::CBX5* (F) *NF1::TJP3* (F) *NTRK1::KIRREL* (F) *PTPRR::CDK4* (F) *ROS1::MYO6* (F) *WHSC1L1::MAK16* (F)
	Extraskeletal myxoid chondrosarcoma	*NR4A3::EWSR1* (F) *EWSR1::NR4A3* (F^d^, G)^c^	
	Ewing sarcoma	*FLI1::EWSR1* (F)^c^ *EWSR1::FLI1* (G)^c^	
	Inflammatory myofibroblastic tumor	*RRBP1::ALK* (F)	
	Intimal sarcoma		*BRCA2::NBEA* (F)^a^ *BRCA2::HS6ST3* (F) *FLT1::TFDP1* (F)
	Leiomyosarcoma		*ALOX12B::CDH13* (F)
	Malignant peripheral nerve sheath tumor		*CNTNAP2::CDKN2A* (F) *IGF1R::CDKN2A* (F)
	Sclerosing epithelioid fibrosarcoma	*EWSR1::CREB3L1* (F)	
	Synovial sarcoma	*SS18::SSX1* (G)^c^	
	Uterine leiomyosarcoma		*FNDC3A::RB1* (F)^b^ *TSC2::PKD1* (F) *NTRK1::KCNH1* (F) *ABL1::NOL8* (F) *ATR::MYLK* (F) *KRAS::BAI3* (F) *FLT1::USPL1* (F)
	Undifferentiated sarcoma		*IQCK::AKT2* (F) *CCND2::NOP2* (F) *TMPRSS2::SFXN1* (F)
	Undifferentiated pleomorphic sarcoma		*PDGFRA::ARHGAP24* (F) *LHFPL3::SMO* (F)
Bone	Chondrosarcoma		*POLD1::ZNF52* (F)
	Osteosarcoma		*NTRK2::CLEC16A* (F) *TSC1::DPYSL3* (F)
	Ewing sarcoma	*EWSR1::FLI1* (G)	
	Pleomorphic sarcoma, undifferentiated		*BCL2::ALPK2* (F)

Abbreviations: F, FoundationOne CDx cancer genome profiling; G, GenMine TOP Cancer Genome Profiling System.

^a^ Previously reported in breast cancer.

^b^ Previously reported in another sarcoma.

^c^ Also detected by reverse transcription polymerase chain reaction.

^d^ Also detected by Fluorescence in situ hybridization.

The TMB values were obtained for all but four patients. The median TMB score was three (range; 0–29). TMB‐H was observed in five patients (3.8%), including two with ULMS (11.7%), one with LMS (17.6%), and one with IMT. TMB‐H was observed in 4/29 (13.7%) and 1/107 (0.9%) patients with ULMS/LMS and other sarcomas, respectively (*p* = 0.007). Microsatellite instability data were available for all but nine patients, and no patient had MSI‐H.

### 3.4 Targetable Genomic Alterations in Sarcoma and Clinical Impact of Genotype‐Matched Therapy

The C‐CAT report described 118 patients (86.8%) with at least 1 oncogenic genomic alteration: 26/36 (72.2%) with TRS, 92/100 (92.0%) with genomically complex sarcomas and other sarcomas (*p* = 0.0073), 19/26 (73.1%) with bone sarcoma, and 99/110 (90.0%) with STS (*p* = 0.047) (Table S12). The C‐CAT report described druggable genomic alterations in 43 patients (level A, 3 patients; level B, 8 patients; level C, 42 patients; and level D, 0 patients). Finally, the expert panel determined that 53 patients (39.0%) had at least 1 druggable genomic alteration that could potentially guide decisions for genotype‐matched therapy (6/28 (21.4%) and 46/108 (42.6%) patients of pediatric/AYA and middle‐aged/older adult (*p* = 0.049), respectively [Table S13]). Twelve patients (8.8%) underwent genotype‐matched therapy, and the efficacy was evaluated in 11 patients (1 patient [7.1%] of pediatric/AYA and 11 patients [10.1%] of middle‐aged/older adult [*p* = 1.00] [Table S14]). Genotype‐matched therapy comprised approved drugs (targeted small‐molecule inhibitors [*N* = 6] and immune checkpoint inhibitors [*N* = 2]), off‐label drugs (targeted small‐molecule inhibitors [*N* = 2] and the other [*N* = 1]), and clinical trials (targeted small‐molecule inhibitors [*N* = 1]) (Table 2). The effect of genotype‐matched therapy was estimated in 11 patients, with a 36.3% response rate (RR) and 81.8% disease control rate (DCR). 1‐year PFS was 31.8% (95% CI: 0.01–0.48) in patients who received genomically matched therapy (Supplementary Figure S1).

**TABLE 2:** Clinical outcome of patients treated with genotype‐matched therapy.

Histology	Number of patients	Targetable alteration	Drug	Best RECIST response	Time to progression (month)	Category (Japan)
LMS	2	High‐TMB	Pembrolizumab	SD, PD	0.9, 6.9	Approved drug
EMC	3	*NR4A3‐EWSR1*	Pazopanib	PR 1, SD 2	43.3 NR (2 cases)	Approved drug
OS	1	*PDGFRA*	Pazopanib	—		Approved drug
OS	1	*NTRK2‐CLEC16A*	Larotrectinib	PD	1.2	Approved drug
MFS	1	*CHEK2*	Cisplatin	PR	4.1	Off‐label drug
IMT	1	*RRBP1‐ALK*	Alectinib	PR	NR	Off‐label drug
DFSP	1	*PDGFRB*	Imatinib	PR	6.2	Off‐label drug
US	1	*PDGFRA*	Pazopanib	PD	3.3	Approved drug
GIST	1	*KIT*	Nilotinib	SD	7.2	Clinical trial (NCCH1901)

Abbreviations: DFSP, dermatofibrosarcoma protuberans; EMC, extraskeletal myxoid chondrosarcoma; EWS, ewing sarcoma of soft tissue; GIST, gastrointestinal stromal tumor; IE, ifosfamide, etoposide; IMT, inflammatory myofibroblastic tumor; LMS, leiomyosarcoma; MFS, myxofibrosarcoma; NR, not reached; OS, osteosarcoma; PD, progressive disease; PR, partial response; SD, stable disease; TMB, tumor mutation burden; US, undifferentiated sarcoma; VDC, vincristine, doxorubicin, cyclophosphamide.

### 3.5 Overall Survival

**FIGURE 3:**
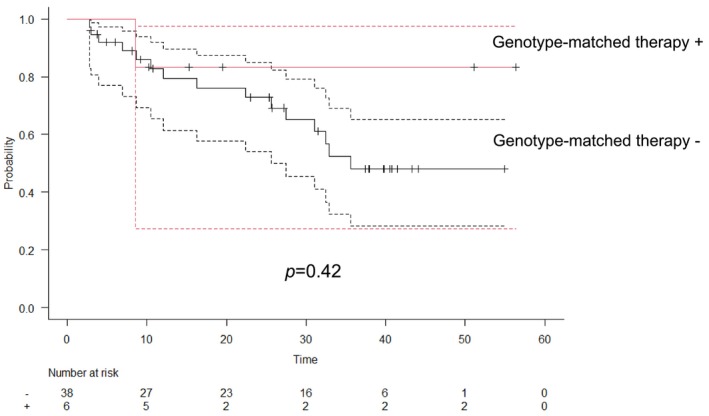
Association between overall survival and the receipt of genomically matched therapy. Patients who received genomically matched therapy had better OS than patients who did not in patients with metastatic or advanced sarcoma who underwent the CGP test before initial systemic treatment: 3‐year OS: 83.3% (95% CI: 0.53–1.0) versus 48.0% (95% CI: 0.53–1.0), *p* = 0.42.

We determined the association between OS and administration of genotype‐matched therapies in patients with metastatic or advanced sarcoma with no prior chemotherapy. There were 44 patients with a median follow‐up of 25 months. Patients who received genomically matched therapy had better OS than patients who did not in patients with metastatic or advanced sarcoma who underwent the CGP test before initial systemic treatment: 3‐year OS: 83.3% (95% CI: 0.53–1.0) vs. 48.0% (95% CI: 0.53–1.0), *p* = 0.42 (Figure 3).

### 3.6 Association Between Overall Survival and Gene Alteration

**FIGURE 4:**
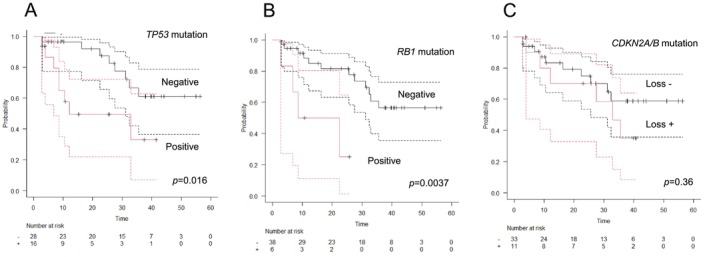
Association between overall survival and gene alteration. Patients with *TP53* (A) and *RB1* (B) mutations but not *CDKN2A* (C) had worse OS than that of patients who did not: 1‐year OS: 96.4% (95% CI: 0.90–1.0) versus 57.7% (95% CI: 0.32–0.84) for *TP53*, *p* = 0.016; 88.3% (95% CI: 0.77–0.99) versus 50.0% (95% CI: 0.10–0.90), *p* = 0.0037 for *RB1*; 83.3% (95% CI: 0.70–0.97) versus 80.0% (95% CI: 0.55–1.0), *p* = 0.36 for *CDKN2A* in patients with metastatic or advanced sarcoma who received CGP test before initial systemic treatment.

Patients with *TP53* and *RB1* mutations but not *CDKN2A* had worse OS than that of patients who did not: 1‐year OS: 96.4% (95% CI: 0.90–1.0) vs. 57.7% (95% CI: 0.32–0.84) for *TP53*, *p* = 0.016; 88.3% (95% CI: 0.77–0.99) vs. 50.0% (95% CI: 0.10–0.90), *p* = 0.0037 for *RB1*; 83.3% (95% CI: 0.70–0.97) vs. 80.0% (95% CI: 0.55–1.0), *p* = 0.36 for *CDKN2A* in patients with metastatic or advanced sarcoma who received CGP test before initial systemic treatment (Figure 4A–C).

### 3.7 Presumed Germline Pathogenic Variants and Germline Pathogenic Variants

Germline findings were detected in 15 patients (11.0%), of which 12 were PGPVs and 3 were GPVs (Table S15). Twelve PGPVs were detected using F1CDx, and two and one GPVs were detected by tumor‐normal matched testing of the NCC Onco‐panel and GenMine TOP, respectively. A total of 16 PGPVs were detected in 12 patients. Seven PGPVs were detected in homologous recombination repair‐related genes, including *BRCA1* (missense: 1 patient), *BRCA2* (loss: 2 patients, rearrangement: 2 patients), and *RAD51C* (loss: 1 patient, rearrangement: 1 patient). PGPVs in disease‐specific genes such as *RB1* (loss: 1 patient, truncating variant: 1 patient), *NF1* (truncating variant: 4 patients, missense: 1 patient), *TP53* (truncating variant: 1 patient), and *SDHA* (missense: 1 patient) were identified in two osteosarcoma cases, one undifferentiated sarcoma case, and three malignant peripheral nerve sheath tumor cases. All the participants were informed of the findings of the study. One patient with a PGPV of *RB1* had a history of retinoblastoma and had already been diagnosed positive for retinoblastoma through confirmatory genetic testing. Three patients with PGPVs of *NF1* had already been diagnosed as neurofibromatosis type 1 by their phenotypes and family history. Three patients underwent confirmatory genetic testing after taking CGPs, followed by streamlined genetic counseling, one of whom was confirmed to have a truly germline origin. In this patient, a single nucleotide variant of NM_007294(*BRCA1*): c.5527G>C (p.A1843P) was identified in *BRCA1* through CGP and was positive for germline origin through genetic testing. The patient was diagnosed with hereditary breast and ovarian cancer syndrome (HBOC), and the patient and family underwent genetic counseling. Regular surveillance of the organs involved was initiated. The unaffected sibling of the patient requested confirmatory genetic testing and was found positive for the same variant in *BRCA1* and subsequently underwent risk‐reducing salpingo‐oophorectomy.

## 4 Discussion

This is an important real‐world study of CGP in the routine clinical setting with important implications for diagnosis and treatment strategies for sarcoma. This study suggests that CGP tests covered by public health insurance could be beneficial for refining diagnosis, treatment decisions, and prognosis prediction in routine clinical settings for sarcoma. Furthermore, the newly emerging dual DNA–RNA panel is useful for detecting histology‐specific fusion genes in sarcomas. Additionally, the germline findings of CGP tests could lead to surveillance and preventive measures for patients and at‐risk relatives.

Boddu et al. revealed that *TP53* (36.8%), *CDKN2A/B* (20.2%), *CDK4/MDM2* (19.3%), *ATRX* (13.2%), and *RB1* (13.2%) were the most frequently detected mutations by CGP tests in patients with advanced or metastatic sarcoma [3]. Consistent with previous reports, this study revealed that the most common gene alterations in patients with sarcoma were *TP53* (36.8%), *RB1* (19.9%), *CDKN2A* (17.6%), *CDK4* (16.2%), and *MDM2* (13.2%). Molecular characteristics (TRS or genomically complex sarcomas/other sarcomas) revealed to be the common factor contributing to the most altered genes (*TP53*, *RB1*, and *CDKN2A*).

Some subtypes are characterized by specific fusion genes that are crucial for correct diagnosis and therapeutic targets [2, 7, 8, 9]. However, the pathological diagnosis of sarcomas remains challenging due to their rarity and morphological complexity, and re‐classification by CGP tests was reported in 4%–10% of patients [3, 5]. In this study, 2.8% of the patients were re‐classified based on the detection of histology‐specific translocations. EMC, characterized by multiple fusion genes, exhibited favorable responses to pazopanib treatment, particularly in patients with *EWSR1::NR4A3* [33]. In this study, histology‐specific fusion genes were identified in nine cases, including *SYT::SSX1* in synovial sarcoma, *EWSR1::FLI1* in ES, and *EWSR1::NR4A3* in EMC. Based on the detection of highly histology‐specific translocations in the sequencing results, three (2.8%) patients were re‐classified. Furthermore, four (2.9%) patients with no prior chemotherapy received chemotherapy based on the histology‐specific fusion gene. Although gene panel testing can contribute to the diagnosis of sarcomas by detecting fusion genes, the detection rate varies depending on the panel used. GenMine TOP, a dual DNA–RNA panel, covers a broad range of fusion genes relevant to sarcomas and thus demonstrated a superior ability to detect histology‐specific fusion genes through RNA analysis [11, 12, 13, 14]. In contrast, traditional DNA‐only CGP tests have limitations in detecting fusion genes, and therefore, their contribution to accurate diagnosis is more restricted compared to GenMine TOP. These differences should be considered when interpreting the diagnostic utility of gene panel testing in sarcomas. In this study, we identified histology‐specific fusion genes in 5 out of 6 TRS cases, whereas other panels detected such fusions in only 5 out of 31 cases (*p* = 0.0035). Indeed, as our study also demonstrated, fusion detection in SFT remains difficult, primarily due to the complex and variable nature of *NAB2*::*STAT6* fusions, which often involve diverse breakpoints and fusion variants that may escape capture by standard panels. We utilized the GenMine TOP DNA–RNA panel to analyze SFT samples included in a large Japanese multi‐institutional database. Notably, we detected the *NAB2::STAT6* fusion in 7 out of 8 SFT cases (unpublished data). This detection rate suggests that GenMine TOP is capable of identifying this diagnostically important fusion in the majority of SFT cases. The clinical utility of GenMine TOP in the diagnosis and treatment planning of sarcomas was revealed. In addition, 33 novel gene fusions involving *RB1*, *BCL2*, and *BRCA2* were identified in 21 patients, predominantly in genomically complex and other sarcomas, not in TRS. However, their functional significance remains unclear, and most of them can be passenger mutations arising during cancer evolution rather than driver mutations.

Previous studies have revealed that the prevalence of TMB‐H and MSI‐H is low in patients with sarcoma (2.6%–4.6% and 0.2%–0.3%, respectively) [8, 34, 35, 36]. This study revealed that 3.8% of patients had TMB‐H. However, heterogeneity was observed, with TMB‐H occurring in 13.7% of ULMS/LMS cases, compared to 0.9% in other sarcomas. Nacev [34] reported considerable heterogeneity, with the median TMB being lowest in well‐differentiated liposarcoma and epithelioid sarcoma, while highest in angiosarcoma (ANGS), UPS, and ULMS, with TMB ≥ 5 mut/Mb in 25% of ANGS and 15% of ULMS and UPS, highlighting the importance of considering the tumor subtype when evaluating TMB‐H in sarcomas.

Targeted therapies and immunotherapy can be new treatment options for sarcomas, which can improve prognosis. Tyrosine kinase inhibitors (TKIs) have remarkable efficacy in various cancers harboring oncogenic fusion genes involving tyrosine kinases [1]. These TKIs include Anaplastic Lymphoma Kinase (ALK) inhibitors, such as crizotinib and alectinib, for ALK fusion‐positive sarcomas and TRK inhibitors, such as larotrectinib and entrectinib, for *NTRK* fusion‐positive sarcomas [1]. In sarcoma, the proportions of potentially druggable alterations and patients who received drugs targeting an alteration detected by CGP were 47%–61% and 11%–16%, respectively [3, 4, 5]. Groisberg et al. reported that 61% of patients had potentially actionable mutations, and 16% of patients received molecular targeted therapy based on NGS results, with an RR of 25% and DCR of 69% [5]. Boddu reported that 49% of patients harbored actionable alterations, 11 received molecular‐targeted therapy, and 1 received immunotherapy (pembrolizumab), with an RR of 8% and a DCR of 17% [3]. Consistent with previous findings, 86.8% and 38.2% of the patients in our cohort study had at least one oncogenic genomic alteration and druggable alteration, respectively. Among them, 13 (9.6%) underwent genotype‐matched therapy based on the advice of the molecular tumor board, including molecular targeted therapy in 9 patients and 2 patients in immunotherapy. The effect of genotype‐matched therapy was fair, with an RR of 36.3% and a DCR of 81.8%. Notably, potentially actionable kinase fusions, including *ALK*, *PDGFRA*, *NTRK1*, *2*, and *ROS1*, were identified in 4.4% of patients, and a patient with IMT harboring *RRBP1::ALK* achieved PR with alectinib treatment. Given the importance of fusion genes in sarcoma management, the combined approach of DNA and RNA analyses of GenMine TOP enables the identification of more fusion genes, which could lead to appropriate molecular targeted therapies for sarcomas.

Hay reported that patients with sarcoma whose treatments were altered by CGP were significantly more likely to have longer PFS (median 124 days) than that of patients whose treatments did not change (median 54 days) [37]. However, the timing of CGP tests during treatment remains unknown [38, 39, 40]. This study is the first to demonstrate the utility of the CGP test in patients with metastatic or advanced sarcoma with no prior chemotherapy. Patients who received genomically matched therapy had better OS than those who did not. Thus, CGP testing before standard chemotherapy may be more effective.

Recently, CGP tests have been reported to play a crucial role in identifying germline findings, with studies indicating that they were detected in 1.7%–15.2% of patients with various malignancies [16, 39]. Kikuchi identified PGPVs in 26 of 171 patients using F1CDx, a tumor‐only test, whereas GPVs were detected in 3 of 18 patients using the NCC Onco‐panel, a paired tumor‐normal matched tests [16]. The hereditary contribution to sarcoma has long been recognized, and several cancer predisposition genes have been implicated, including *TP53*, *BRCA1/2*, *RB1*, and *NF1* [16]. Then, it is likely that some of the patients with PGPVs/GPVs carry underlying hereditary cancer syndromes such as neurofibromatosis type 1 (NF1) or Li‐Fraumeni syndrome. However, reports on the detection rates of germline pathogenic variants using CGP in real‐world sarcoma cases remain limited. The tumor‐only CGP panels, such as F1CDx, cannot reliably distinguish germline from somatic mutations. In contrast, NCC Onco‐panel and GenMine TOP incorporate tumor–normal matching design and identify GPVs. In this study, PGPVs/GPVs were identified in 11.8% of patients, with most mutations found in genes related to homologous recombination repair (*BRCA1*, *BRCA2* and *RAD51C*) and disease‐specific genes (*RB1* and *NF1*). These findings have important implications in the management of patients and at‐risk family members. As demonstrated, the diagnosis of HBOC by the detection of *a BRCA1* mutation through CGP resulted in regular surveillance of the patient and her family. Thus, patients identified with hereditary cancer syndromes by CGP should receive proper genetic counseling, enabling patients and their at‐risk relatives to perform personalized cancer management, including surveillance and preventive measures. An additional advantage of detecting germline mutations is their relevance to targeted therapies. Germline defects in homologous recombination repair genes, such as *BRCA1* and *BRCA2*, are predictive biomarkers for response to PARP inhibitors [41]. Therefore, integrating germline testing into clinical cancer management is increasingly necessary to guide both therapeutic strategies and genetic counseling.

This study had several limitations. First, this is a single‐center study using a limited number of cases with histological heterogeneity, and some of the subtypes included only one or two individuals. Thus, future studies using more patients are needed. Second, there was selection bias in cases in which a CGP panel was performed because this was a retrospective study. There is a need to examine the impact of the CGP panel on clinical practice by prospectively examining all sarcoma cases over a period to examine the usefulness of the CGP panel. Third, the availability of clinical trials may differ depending on when the CGP test is performed; therefore, the recommended candidate drugs may differ. Fourth, patients who received genomically matched therapy had better overall survival than those who did not. However, these findings are limited to this specific patient with metastatic or advanced sarcoma who has not received prior chemotherapy and may not apply to all patients undergoing CGP testing. At the one‐year time point, it is likely that later‐line treatments did not yet show an impact on long‐term OS. Fifth, in our cohort, it is indeed challenging to completely rule out the possibility of clonal hematopoiesis, particularly for the variant, which showed low VAF and lacked clear phenotypic manifestations.

## 5 Conclusion

In conclusion, publicly reimbursed CGP tests, particularly the dual DNA–RNA panel, contribute to refined diagnostic precision in selected sarcoma subtypes and inform critical treatment decisions. Although the overall frequency of diagnosis‐altering fusion detection was limited, the DNA–RNA panel significantly outperformed DNA‐only panels in identifying histotype‐defining translocations (*p* = 0.0035), enabling molecular reclassification in cases where conventional diagnostics were inconclusive. The implementation of genotype‐matched therapies showed favorable clinical outcomes and improved the prognosis. CGP tests also identified germline findings, enabling patients and their at‐risk relatives to perform personalized cancer management, including surveillance and preventive measures.

## Author Contributions

**Eiji Nakata:** conceptualization, investigation, writing – original draft, writing – review and editing, methodology. **Daisuke Ennishi:** conceptualization, investigation, writing – original draft. **Tatsunori Osone:** investigation, writing – original draft. **Kiichiro Ninomiya:** conceptualization, writing – original draft, investigation. **Shuta Tomida:** conceptualization, writing – original draft, investigation. **Takuto Itano:** conceptualization, writing – original draft, investigation. **Tomohiro Fujiwara:** conceptualization, investigation, writing – original draft. **Toshiyuki Kunisada:** conceptualization, funding acquisition, investigation, writing – original draft. **Naoyuki Ida:** conceptualization, investigation, writing – original draft. **Hideki Yamamoto:** conceptualization, investigation, writing – original draft. **Mashu Futagawa:** conceptualization, investigation, writing – original draft. **Tatsunori Shimoi:** conceptualization, writing – original draft. **Hiroyuki Yanai:** conceptualization, investigation, writing – original draft. **Akira Hirasawa:** conceptualization, investigation, writing – original draft. **Shinichi Toyooka:** conceptualization, writing – original draft, investigation. **Masahiro Tabata:** conceptualization, investigation, writing – original draft. **Toshifumi Ozaki:** conceptualization, investigation, writing – original draft.

## Ethics Statement

This retrospective chart review study involving human participants was in accordance with the ethical standards of the institutional and national research committee and with the 1964 Helsinki Declaration and its later amendments or comparable ethical standards. The Human Investigation Committee (IRB) of Okayama University Hospital approved this study (approval number K2111‐047).

## Consent

Written informed consent was obtained from each participant included in this study.

## Conflicts of Interest

The authors declare no conflicts of interest.

## Supporting information


**Figure S1:** Progression‐free survival (PFS). 1‐year PFS was 14.3% in patients who received genomically matched therapy.

**Table S1:** cam471098‐sup‐0002‐TableS1.docx.

**Table S2:** cam471098‐sup‐0003‐TableS2.docx.

**Table S3:** cam471098‐sup‐0004‐TableS3.xlsx.

**Table S4:** cam471098‐sup‐0005‐TableS4.docx.

**Table S5:** cam471098‐sup‐0006‐TableS5.docx.

**Table S6:** cam471098‐sup‐0007‐TableS6.docx.

**Table S7:** cam471098‐sup‐0008‐TableS7.xlsx.

**Table S8:** cam471098‐sup‐0009‐TableS8.docx.

**Table S9:** cam471098‐sup‐0010‐TableS9.docx.

**Table S10:** cam471098‐sup‐0011‐TableS10.docx.

**Table S11:** cam471098‐sup‐0012‐TableS11.docx.

**Table S12:** cam471098‐sup‐0013‐TableS12.docx.

**Table S13:** cam471098‐sup‐0014‐TableS13.docx.

**Table S14:** cam471098‐sup‐0015‐TableS14.docx.

**Table S15:** cam471098‐sup‐0016‐TableS15.xlsx.

## Data Availability

The data that support the findings of this study are available from the corresponding author upon reasonable request.
