# Supplementary figures and images for: Real‐World Data of Comprehensive Cancer Genomic Profiling Tests Performed in the Routine Clinical Setting in Sarcoma
Source: Cancer Med. 2025 Aug 4;14(15):e71098. doi: 10.1002/cam4.71098 (PMC12320126; doi:10.1002/cam4.71098)

## Slide 1
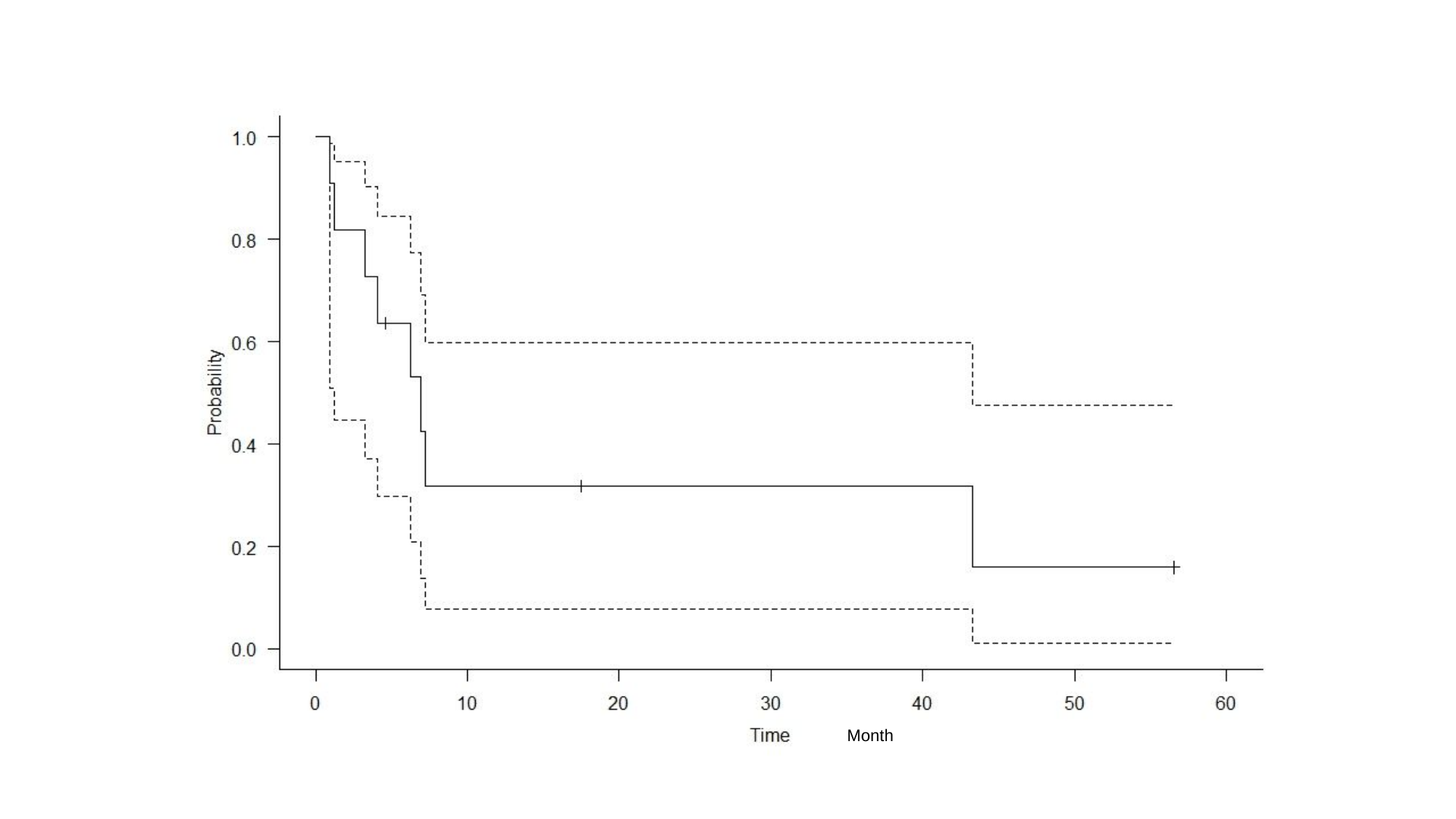

Month

Supplement: Supplementary file 1 — Figure S1: Progression‐free survival (PFS). 1‐year PFS was 14.3% in patients who received genomically matched therapy. [file CAM4-14-e71098-s003.ppt]
