# Supplementary material for: Real‐World Data of Comprehensive Cancer Genomic Profiling Tests Performed in the Routine Clinical Setting in Sarcoma
Source: Cancer Med. 2025 Aug 4;14(15):e71098. doi: 10.1002/cam4.71098 (PMC12320126; doi:10.1002/cam4.71098)
Supplement: Supplementary file 2 — Table S1: cam471098‐sup‐0002‐TableS1.docx. [file CAM4-14-e71098-s016.docx]

**Supplementary Table1.** **Categorization of sarcoma types**

| Characteristic | Translocation-related sarcoma | Genomically complex and other sarcoma |
| --- | --- | --- |
| Soft tissue sarcoma | Alveolar rhabdomyosarcoma | Angiosarcoma |
|  | Alveolar soft part sarcoma | Dedifferentiated liposarcoma |
|  | Sarcoma with *BCOR* genetic alterations | Epithelioid sarcoma |
|  | Dermatofibrosarcoma protuberans | Extraskeletal osteosarcoma |
|  | Extraskeletal myxoid chondrosarcoma | Gastrointestinal stromal tumor |
|  | Ewing sarcoma of soft tissue | Intimal sarcoma |
|  | Inflammatory myofibroblastic tumor | Leiomyosarcoma |
|  | Myxoid/round cell liposarcoma | Malignant peripheral nerve sheath tumor |
|  | Sclerosing epithelioid fibrosarcoma | Myxofibrosarcoma |
|  | Solitary fibrous tumor | Pleomorphic rhabdomyosarcoma |
|  | Synovial sarcoma | Undifferentiated sarcoma |
|  |  | Uterine leiomyosarcoma |
| Bone sarcoma | Ewing sarcoma | Osteosarcoma |
|  | Epithelioid hemangioendothelioma | Chondrosarcoma |
|  | Mesenchymal chondrosarcoma | Chordoma |
|  |  | Pleomorphic sarcoma, undifferentiated |
|  |  | Leiomyosarcoma |
|  |  | Malignant giant cell tumor of bone |
