# Supplementary material for: Real‐World Data of Comprehensive Cancer Genomic Profiling Tests Performed in the Routine Clinical Setting in Sarcoma
Source: Cancer Med. 2025 Aug 4;14(15):e71098. doi: 10.1002/cam4.71098 (PMC12320126; doi:10.1002/cam4.71098)
Supplement: Supplementary file 3 — Table S2: cam471098‐sup‐0003‐TableS2.docx. [file CAM4-14-e71098-s015.docx]

**Supplementary Table 2. Comprehensive cancer genomic profiling tests**

| Characteristic | OncoGuide™ NCC Oncopanel System | FoundationOne^®^ CDx cancer genome profiling | FoundationOne^®^ Liquid CDx cancer genome profiling | GenMineTOP™ Cancer Genome Profiling System |
| --- | --- | --- | --- | --- |
| Specimen | FFPE  Peripheral blood | FFPE | Peripheral blood | FFPE  Peripheral blood |
| DNA | 124 genes | 324 genes | 324 genes | 737 genes |
| RNA | - | - | - | 27 genes |
| Fusion gene | 13 genes | 36 genes | 36 genes | 455 genes |

FFPE; Formalin-fixed paraffin-embedded samples
