# Supplementary material for: Real‐World Data of Comprehensive Cancer Genomic Profiling Tests Performed in the Routine Clinical Setting in Sarcoma
Source: Cancer Med. 2025 Aug 4;14(15):e71098. doi: 10.1002/cam4.71098 (PMC12320126; doi:10.1002/cam4.71098)
Supplement: Supplementary file 5 — Table S4: cam471098‐sup‐0005‐TableS4.docx. [file CAM4-14-e71098-s014.docx]

**Supplementary table 4. Evidence levels based on clinical practice guidance for NGS in cancer diagnosis and treatment (Edition 2.0)**

| Evidence levels | Evidence level classifications |
| --- | --- |
| A | Genetic abnormality that predicts response to FDA or PMDA-approved therapies for a specific type of tumor  Biomarkers included in professional guidelines as predicting factors for a specific type of tumor |
| B | Biomarkers that predict responses to therapies for a specific type of tumor based on well-powered studies with consensus from experts in the field |
| C | Biomarkers that predict responses to therapies approved by the PMDA or FDA for a different type of tumor  Biomarkers of therapeutic significance based on the results of small studies  Biomarkers that predict responses to therapies for a different type of tumor based on well-powered studies with consensus from experts in the field |
| D | Biomarkers associated with efficacy in a few case reports |
| E | Biomarkers that have plausible therapeutic significance based on preclinical studies |
| F | Gene abnormality known to be involved in cancer |
| R | Known to be related to drug resistance |

NGS; Next-Generation Sequencing

FDA; Food and Drug Administration

PMDA; Pharmaceuticals and Medical Devices Agency
