# Supplementary material for: Real‐World Data of Comprehensive Cancer Genomic Profiling Tests Performed in the Routine Clinical Setting in Sarcoma
Source: Cancer Med. 2025 Aug 4;14(15):e71098. doi: 10.1002/cam4.71098 (PMC12320126; doi:10.1002/cam4.71098)
Supplement: Supplementary file 6 — Table S5: cam471098‐sup‐0006‐TableS5.docx. [file CAM4-14-e71098-s007.docx]

**Supplementary Table 5. Patient characteristics**

| Characteristics | Category | Patients, number |
| --- | --- | --- |
| Sex | Male | 62 |
|  | Female | 74 |
| Age, year | Median (range) | 55 (8–80 years) |
| Generation | Pediatric | 5 |
|  | Adolescent and Young Adult | 23 |
|  | Middle-aged | 62 |
|  | Older adult | 46 |
| Primary tumor site |  |  |
| Soft tissue | Head & neck | 11 |
|  | Thoracic | 5 |
|  | Trunk | 6 |
|  | Retroperitoneal | 18 |
|  | Intrabdominal | 17 |
|  | Visceral | 3 |
|  | Uterine | 17 |
|  | Extremity | 33 |
| Bone | Vertebral body | 6 |
|  | Pelvis | 2 |
|  | Skull | 2 |
|  | Rib | 1 |
|  | Femur | 8 |
|  | Fibula | 2 |
|  | Humerus | 5 |
| CGP test | F1CDx | 118 |
|  | GenMine TOP | 12 |
|  | NCC Oncopanel | 4 |
|  | F1LCDx | 2 |
| Site of specimen |  |  |
| FFPE | Primary site | 75 |
|  | Local recurrent site | 15 |
|  | Metastatic site | 44 |
| Peripheral blood |  | 2 |
| Sampling methods | Surgery | 103 |
|  | Biopsy | 30 |
|  | Liquid | 2 |
|  | Cell block from pleural effusion | 1 |
| Prior chemotherapy | 0 | 43 |
|  | 1 | 53 |
|  | 2 | 15 |
|  | 3 | 16 |
|  | 4 | 5 |
|  | 5 | 4 |

CGP; comprehensive cancer genomic profiling

F1CDx; FoundationOne^®^ CDx cancer genome profiling

GenMine TOP; GenMine TOP^®^ Cancer Genome Profiling System

NCC Oncopanel; OncoGuide™ NCC Oncopanel System

F1LCDx; FoundationOne^®^ Liquid CDx cancer genome profiling ]

FFPE; Formalin-fixedparaffin-embedded samples
