# Supplementary material for: Real‐World Data of Comprehensive Cancer Genomic Profiling Tests Performed in the Routine Clinical Setting in Sarcoma
Source: Cancer Med. 2025 Aug 4;14(15):e71098. doi: 10.1002/cam4.71098 (PMC12320126; doi:10.1002/cam4.71098)
Supplement: Supplementary file 7 — Table S6: cam471098‐sup‐0007‐TableS6.docx. [file CAM4-14-e71098-s008.docx]

**Supplementary Table 6. Histology of the patients**

| Characteristics | Category | Patients, number |
| --- | --- | --- |
| Soft tissue sarcoma | Dedifferentiated liposarcoma | 19 |
|  | Uterine leiomyosarcoma | 17 |
|  | Leiomyosarcoma | 12 |
|  | Myxoid/round cell liposarcoma | 10 |
|  | Undifferentiated sarcoma | 10 |
|  | Malignant peripheral nerve sheath tumor | 7 |
|  | Myxofibrosarcoma | 4 |
|  | Synovial sarcoma | 4 |
|  | Alveolar soft part sarcoma | 4 |
|  | Extraskeletal myxoid chondrosarcoma | 3 |
|  | Solitary fibrous tumor | 3 |
|  | Alveolar rhabdomyosarcoma | 2 |
|  | Dermatofibrosarcoma protuberans | 2 |
|  | Epithelioid sarcoma | 2 |
|  | Inflammatory myofibroblastic tumor | 2 |
|  | Extraskeletal osteosarcoma | 2 |
|  | Angiosarcoma | 1 |
|  | Sarcoma with *BCOR* genetic alterations | 1 |
|  | Ewing sarcoma | 1 |
|  | Gastrointestinal stromal tumor | 1 |
|  | Intimal sarcoma | 1 |
|  | Sclerosing epithelioid fibrosarcoma | 1 |
|  | Pleomorphic rhabdomyosarcoma | 1 |
| Bone sarcoma | Osteosarcoma | 11 |
|  | Chondrosarcoma | 5 |
|  | Chordoma | 4 |
|  | Ewing sarcoma | 1 |
|  | Leiomyosarcoma | 1 |
|  | Mesenchymal chondrosarcoma | 1 |
|  | Pleomorphic sarcoma, undifferentiated | 1 |
|  | Epithelioid hemangioendothelioma | 1 |
|  | Malignant giant cell tumor of bone | 1 |
