# Supplementary material for: Real‐World Data of Comprehensive Cancer Genomic Profiling Tests Performed in the Routine Clinical Setting in Sarcoma
Source: Cancer Med. 2025 Aug 4;14(15):e71098. doi: 10.1002/cam4.71098 (PMC12320126; doi:10.1002/cam4.71098)
Supplement: Supplementary file 9 — Table S8: cam471098‐sup‐0009‐TableS8.docx. [file CAM4-14-e71098-s001.docx]

**Supplementary Table 8. Associated factors of gene mutation of *TP53***

AYA; adolescent and young adult

| Variable | Category | Patients, number (%) | | p-Value |
| --- | --- | --- | --- | --- |
|  |  | Patients with  gene mutation of *TP53* | Patients without  gene mutation of *TP53* |  |
| Generation | Pediatric/AYA | 5 (17.9%) | 23 (82.1%) | 0.027 |
|  | Middle-aged/older adult | 45 (41.7%) | 63 (58.3%) |  |
|  |  |  |  |  |
| Sex | Male | 19 (30.6%) | 43 (69.4%) | 0.21 |
|  | Female | 31 (41.9%) | 43 (58.1%) |  |
|  |  |  |  |  |
| Primary tumor | Yes | 26 (34.7%) | 49 (65.3%) | 0.60 |
|  | No | 24 (39.3%) | 37 (60.7%) |  |
|  |  |  |  |  |
| Genomic character | Translocation-related sarcomas | 4 (12.5%) | 32 (87.5%) | P < 0.001 |
|  | Genomically complex and other sarcomas | 46 (46.0%) | 54 (54.0%) |  |
|  |  |  |  |  |
| Originated tissue | Bone | 4 (15.4%) | 22 (84.6%) | 0.013 |
|  | Soft tissue | 46 (41.8%) | 64 (58.2%) |  |
