# Supplementary material for: Real‐World Data of Comprehensive Cancer Genomic Profiling Tests Performed in the Routine Clinical Setting in Sarcoma
Source: Cancer Med. 2025 Aug 4;14(15):e71098. doi: 10.1002/cam4.71098 (PMC12320126; doi:10.1002/cam4.71098)
Supplement: Supplementary file 10 — Table S9: cam471098‐sup‐0010‐TableS9.docx. [file CAM4-14-e71098-s005.docx]

**Supplementary Table 9. Associated factors of gene mutation of *RB1***

| Variable | Category | Patients, number (%) | | p-Value |
| --- | --- | --- | --- | --- |
|  |  | Patients with  gene mutation of *RB1* | Patients without  gene mutation of *RB1* |  |
| Generation | Pediatric/AYA | 3 (10.7%) | 25 (89.3%) | 0.29 |
|  | Middle-aged/older adult | 24 (22.2%) | 84 (77.8%) |  |
|  |  |  |  |  |
| Sex | Male | 7 (11.3%) | 55 (88.7%) | 0.030 |
|  | Female | 20 (27.0%) | 54 (73.0%) |  |
|  |  |  |  |  |
| Primary tumor | Yes | 12 (16.0%) | 63 (84.0%) | 0.28 |
|  | No | 15 (24.6%) | 46 (75.4%) |  |
|  |  |  |  |  |
| Genomic character | Translocation-related sarcomas | 1 (2.8%) | 35 (97.2%) | P < 0.001 |
|  | Genomically complex and other sarcomas | 26 (46.0%) | 74 (54.0%) |  |
|  |  |  |  |  |
| Originated tissue | Bone | 3 (11.5%) | 23 (88.5%) | 0.29 |
|  | Soft tissue | 24 (21.8%) | 86 (78.2%) |  |

AYA; adolescent and young adult
