# Supplementary material for: Real‐World Data of Comprehensive Cancer Genomic Profiling Tests Performed in the Routine Clinical Setting in Sarcoma
Source: Cancer Med. 2025 Aug 4;14(15):e71098. doi: 10.1002/cam4.71098 (PMC12320126; doi:10.1002/cam4.71098)
Supplement: Supplementary file 11 — Table S10: cam471098‐sup‐0011‐TableS10.docx. [file CAM4-14-e71098-s013.docx]

**Supplementary Table 10. Associated factors of gene mutation of *CDKN2A***

| Variable | Category | Patients, number | | p-Value |
| --- | --- | --- | --- | --- |
|  |  | Patients with  gene mutation of *CDKN2A* | Patients without  gene mutation of *CDKN2A* |  |
| Generation | Pediatric/AYA | 5 (17.9%) | 23 (82.1%) | 1.00 |
|  | Middle-aged/older adult | 19 (17.6%) | 89 (82.4%) |  |
|  |  |  |  |  |
| Sex | Male | 9 (14.5%) | 53 (85.5%) | 0.50 |
|  | Female | 15 (20.3%) | 59 (79.7%) |  |
|  |  |  |  |  |
| Primary tumor | Yes | 12 (16.0%) | 63 (84.0%) | 0.65 |
|  | No | 12 (19.7%) | 49 (80.3%) |  |
|  |  |  |  |  |
| Genomic character | Translocation-related sarcomas | 2 (5.6%) | 34 (94.4%) | 0.039 |
|  | Genomically complex and other sarcomas | 22 (22.0%) | 78 (78.0%) |  |
|  |  |  |  |  |
| Originated tissue | Bone | 8 (30.8%) | 18 (69.2%) | 0.082 |
|  | Soft tissue | 16 (14.5%) | 94 (85.5%) |  |

AYA; adolescent and young adult

*CDKN2A*; cyclin-dependent kinase inhibitor 2A
