# Supplementary material for: Real‐World Data of Comprehensive Cancer Genomic Profiling Tests Performed in the Routine Clinical Setting in Sarcoma
Source: Cancer Med. 2025 Aug 4;14(15):e71098. doi: 10.1002/cam4.71098 (PMC12320126; doi:10.1002/cam4.71098)
Supplement: Supplementary file 12 — Table S11: cam471098‐sup‐0012‐TableS11.docx. [file CAM4-14-e71098-s009.docx]

**Supplementary Table 11. The difference of the detection of common recurrent fusion by CGP test**

| Histology of translocation-related sarcomas | Common Recurrent fusion | Patients, number | |
| --- | --- | --- | --- |
|  |  | GenMine TOP | Others |
| Alveolar rhabdomyosarcoma | *PAX3::FOXO1*  *PAX7::FOXO1* | - | 0 (2) |
| Alveolar soft part sarcoma | *ASPSCR1::TFE3* | 1 (1) | 0 (3) |
| Sarcoma with *BCOR* genetic alterations | *BCOR::CCNB3* | 1 (1) | - |
| Dermatofibrosarcoma protuberans | *COL1A1::PDGFB* | - | 0 (2) |
| Epithelioid hemangioendothelioma | *WWTR1::CAMTA1* |  | 0 (1) |
| Extraskeletal myxoid chondrosarcoma | *NR4A3::EWSR1*  *EWSR1::NR4A3* | 1 (1) | 2 (2) |
| Ewing sarcoma | *EWSR1::FLI1*  *FLI1::EWSR1* | 1 (1) | 1 (1) |
| Inflammatory myofibroblastic tumor | *TPM3::ALK*  *RRBP1::ALK* |  | 1 (2) |
| Mesenchymal chondrosarcoma | *HEY1-NCOA2* | - | 0 (1) |
| Myxoid/round cell liposarcoma | *FUS::DDIT3*  *EWSR1::DDIT3* | 0 (1) | 0 (9) |
| Sclerosing epithelioid fibrosarcoma | *EWSR1::CREB3L1* | - | 1 (1) |
| Solitary fibrous tumor | *NAB2::STAT6* | - | 0 (3) |
| Synovial sarcoma | *SS18::SSX1, 2* | 1 (1) | 0 (3) |

CGP; comprehensive cancer genomic profiling

GenMine TOP; GenMine TOP^®^ Cancer Genome Profiling System
