# Supplementary material for: Real‐World Data of Comprehensive Cancer Genomic Profiling Tests Performed in the Routine Clinical Setting in Sarcoma
Source: Cancer Med. 2025 Aug 4;14(15):e71098. doi: 10.1002/cam4.71098 (PMC12320126; doi:10.1002/cam4.71098)
Supplement: Supplementary file 13 — Table S12: cam471098‐sup‐0013‐TableS12.docx. [file CAM4-14-e71098-s011.docx]

**Supplementary Table 12. Associated factors of** **oncogenic gene mutation**

AYA; adolescent and young adult

| Variable | Category | Patients, number | | p-Value |
| --- | --- | --- | --- | --- |
|  |  | Patients with  oncogenic gene mutation | Patients without  oncogenic gene mutation |  |
| Generation | Pediatric/AYA | 22 (78.6%) | 6 (21.4%) | 0.21 |
|  | Middle-aged/older adult | 96 (88.9%) | 12 (11.1%) |  |
|  |  |  |  |  |
| Sex | Male | 54 (87.1%) | 8 (12.9%) | 1.00 |
|  | Female | 64 (86.5%) | 10 (13.5%) |  |
|  |  |  |  |  |
| Primary tumor | Yes | 66 (88.0%) | 9 (12.0%) | 0.80 |
|  | No | 52 (85.2%) | 9 (14.8%) |  |
|  |  |  |  |  |
| Genomic character | Translocation-related sarcomas | 26 (72.2%) | 10 (27.8%) | 0.0073 |
|  | Genomically complex and other sarcomas | 92 (92.0%) | 8 (8.0%) |  |
|  |  |  |  |  |
| Originated tissue | Bone | 19 (73.1%) | 7 (26.9%) | 0.047 |
|  | Soft tissue | 99 (90.0%) | 11 (10.0%) |  |
