# Supplementary material for: Real‐World Data of Comprehensive Cancer Genomic Profiling Tests Performed in the Routine Clinical Setting in Sarcoma
Source: Cancer Med. 2025 Aug 4;14(15):e71098. doi: 10.1002/cam4.71098 (PMC12320126; doi:10.1002/cam4.71098)
Supplement: Supplementary file 14 — Table S13: cam471098‐sup‐0014‐TableS13.docx. [file CAM4-14-e71098-s006.docx]

**Supplementary Table 13. Associated factors of druggable gene mutation**

| Variable | Category | Patients, number | | p-Value |
| --- | --- | --- | --- | --- |
|  |  | Patients with druggable gene mutation | Patients without  druggable gene mutation |  |
| Generation | Pediatric/AYA | 6 | 22 | 0.049 |
|  | Middle-aged/older adult | 46 | 62 |  |
|  |  |  |  |  |
| Sex | Male | 27 | 35 | 0.29 |
|  | Female | 25 | 49 |  |
|  |  |  |  |  |
| Primary tumor | Yes | 26 | 49 | 0.38 |
|  | No | 26 | 35 |  |
|  |  |  |  |  |
| Genomic character | Translocation-related sarcomas | 9 | 27 | 0.072 |
|  | Genomically complex and other sarcomas | 43 | 57 |  |
|  |  |  |  |  |
| Originated tissue | Bone | 6 | 20 | 0.12 |
|  | Soft tissue | 46 | 64 |  |

AYA; adolescent and young adult
