# Supplementary material for: Real‐World Data of Comprehensive Cancer Genomic Profiling Tests Performed in the Routine Clinical Setting in Sarcoma
Source: Cancer Med. 2025 Aug 4;14(15):e71098. doi: 10.1002/cam4.71098 (PMC12320126; doi:10.1002/cam4.71098)
Supplement: Supplementary file 15 — Table S14: cam471098‐sup‐0015‐TableS14.docx. [file CAM4-14-e71098-s012.docx]

**Supplementary Table 14. Factors associated with genotype-matched therapy**

AYA; adolescent and young adult

| Variable | Category | Patients, number | | p-Value |
| --- | --- | --- | --- | --- |
|  |  | Patients with genotype-matched therapy | Patients without genotype-matched therapy |  |
| Generation | Pediatric/AYA | 2 | 26 | 1.00 |
|  | Middle-aged/older adult | 10 | 98 |  |
|  |  |  |  |  |
| Sex | Male | 8 | 54 | 0.14 |
|  | Female | 4 | 70 |  |
|  |  |  |  |  |
| Primary tumor | Yes | 7 | 66 | 1.00 |
|  | No | 5 | 56 |  |
|  |  |  |  |  |
| Genomic character | Translocation-related sarcomas | 5 | 31 | 0.30 |
|  | Genomically complex and other sarcomas | 7 | 93 |  |
|  |  |  |  |  |
| Originated tissue | Bone | 2 | 24 | 1.00 |
|  | Soft tissue | 10 | 100 |  |
